# Human Prion Diseases in The Netherlands (1998–2009): Clinical, Genetic and Molecular Aspects

**DOI:** 10.1371/journal.pone.0036333

**Published:** 2012-04-30

**Authors:** Casper Jansen, Piero Parchi, Sabina Capellari, Carla A. Ibrahim-Verbaas, Maaike Schuur, Rosaria Strammiello, Patrizia Corrado, Matthew T. Bishop, Willem A. van Gool, Marcel M. Verbeek, Frank Baas, Wesley van Saane, Wim G. M. Spliet, Gerard H. Jansen, Cornelia M. van Duijn, Annemieke J. M. Rozemuller

**Affiliations:** 1 Dutch Surveillance Centre for Prion Diseases, University Medical Centre Utrecht, Utrecht, The Netherlands; 2 Istituto delle Scienze Neurologiche and Dipartimento di Scienze Neurologiche, Università di Bologna, Bologna, Italy; 3 Department of Neurology, Erasmus University Medical Centre, Rotterdam, The Netherlands; 4 Dutch National Prion Disease Registry, Department of Epidemiology, Erasmus University Medical Center, Rotterdam, The Netherlands; 5 National Creutzfeldt-Jakob Disease Surveillance Unit, University of Edinburgh, Edinburgh, United Kingdom; 6 Department of Neurology, Academic Medical Centre, Amsterdam, The Netherlands; 7 Radboud University Nijmegen Medical Centre, Departments of Neurology and Laboratory Medicine, Donders Institute for Brain Cognition and Behavior, Alzheimer Centre Nijmegen, Nijmegen, The Netherlands; 8 Department of Genome Analysis, Academic Medical Centre, Amsterdam, The Netherlands; 9 Creutzfeldt-Jakob Disease Surveillance System, Prion Diseases Program, Public Health Agency of Canada, Ottawa, Ontario, Canada; 10 Netherlands Brain Bank, Amsterdam, The Netherlands; 11 Department of Pathology, VU University Medical Center, Amsterdam, The Netherlands; Oslo University Hospital, Norway

## Abstract

Prion diseases are rare and fatal neurodegenerative disorders that can be sporadic, inherited or acquired by infection. Based on a national surveillance program in the Netherlands we describe here the clinical, neuropathological, genetic and molecular characteristics of 162 patients with neuropathologically confirmed prion disease over a 12-year period (1998–2009). Since 1998, there has been a relatively stable mortality of Creutzfeldt-Jakob disease (CJD) in the Netherlands, ranging from 0.63 to 1.53 per million inhabitants per annum. Genetic analysis of the codon 129 methionine/valine (M/V) polymorphism in all patients with sporadic CJD (sCJD) showed a trend for under-representation of VV cases (7.0%), compared with sCJD cohorts in other Western countries, whereas the MV genotype was relatively over-represented (22,4%). Combined PrP^Sc^ and histopathological typing identified all sCJD subtypes known to date, except for the VV1 subtype. In particular, a “pure" phenotype was demonstrated in 60.1% of patients, whereas a mixed phenotype was detected in 39.9% of all sCJD cases. The relative excess of MV cases was largely accounted for by a relatively high incidence of the MV 2K subtype. Genetic analysis of the prion protein gene (*PRNP*) was performed in 161 patients and showed a mutation in 9 of them (5.6%), including one FFI and four GSS cases. Iatrogenic CJD was a rare phenomenon (3.1%), mainly associated with dura mater grafts. Three patients were diagnosed with new variant CJD (1.9%) and one with variably protease-sensitive prionopathy (VPSPr). Post-mortem examination revealed an alternative diagnosis in 156 patients, most commonly Alzheimer's disease (21.2%) or vascular causes of dementia (19.9%). The mortality rates of sCJD in the Netherlands are similar to those in other European countries, whereas iatrogenic and genetic cases are relatively rare. The unusual incidence of the VV2 sCJD subtype compared to that reported to date in other Western countries deserves further investigation.

## Introduction

Prion diseases, also known as transmissible spongiform encephalopathies (TSEs), are fatal neurodegenerative disorders that occur in both humans and a wide variety of animals, such as cattle, sheep, deer and elk [1]. The most common form in humans, accounting for 85–90% of all prion diseases, is sporadic Creutzfeldt-Jakob disease (sCJD), which has an unknown aetiology and occurs with a worldwide annual incidence of 1–1,5 per million [2], [3]. Familial forms make up 5–15% of the total number of cases and follow a dominant mode of inheritance. They are caused by pathogenic mutations in *PRNP*, the gene encoding the prion protein [4]. Over 30 different mutations have been described to date, resulting in clinico-pathological entities such as genetic CJD (gCJD), Gerstmann-Straüssler-Scheinker (GSS) disease and Fatal Familial Insomnia (FFI) [5], [6]. Finally, a small proportion of cases (1–2%) are acquired by infection. The majority of these have been transmitted through medical procedures (e.g. in pituitary hormone and dura mater grafts recipients), thus the designation of iatrogenic CJD (iCJD) [7].

The central event in prion diseases is the conversion of the normal, physiological form of the prion protein (PrP^C^) into a misfolded and aggregated β-sheet rich form, commonly referred to as PrP^Sc^ [1]. This conversion leads to the formation of several PrP^Sc^ “types" that can be distinguished on the basis of physicochemical properties such as the size of the fragment resistant to treatment with proteinase K (PK), possibly reflecting distinct protein conformations, and the relative abundance of the three differently glycosylated isoforms of the protein (i.e. glycoform ratio). Based on the size of the core fragment (i.e. PrP27–30) generated by PK digestion, Parchi *et al.* were able to identify two major PrP^Sc^ types: type 1 with a relative molecular mass of 21 kDa and type 2 with a relative molecular mass of 19 kDa [8]. Evidence indicates that the formation of these two major PrP^Sc^ types is in part influenced by the *PRNP* genotype, especially the codon 129 methionine/valine (M/V) polymorphism; whilst type 1 is the most common finding in MM subjects, type 2 largely predominates in subjects carrying VV.

One of the major characteristics of human prion diseases is the heterogeneity of the clinical and pathological phenotype [9]. Prion strains, originally defined by their distinct phenotypes upon transmission to syngenic animals, which are maintained on serial transmission, are believed to be the main cause of this phenotypic variability [10]. In addition, the host genotype variability in *PRNP*, as determined by polymorphisms and mutations, is another recognized causal factor for phenotypic heterogeneity [11]–[13]. There is now general consensus that in sCJD the M/V genotype at codon 129 of *PRNP*, the type of PrP^Sc^, as well as the ratio of the PrP^Sc^ glycoforms, are major determinants of the disease phenotype and can serve as surrogate “strain-typing" markers. The combination of the codon 129 genotype and the PrP^Sc^ type has allowed for the distinction of six subtypes of sCJD, each with a characteristic incidence, and distinctive clinical and histopathological features, that are classified as (i) MM1/MV1; (ii) VV2; (iii) MV 2K; (iv) MM 2C; (v) MM 2T or sporadic FI; and (vi) VV1 [13], [14]. Although much remains to be learned about the biological basis of prion strains, recent studies have identified at least five distinct strains in humans that correspond to most of the sCJD subtypes listed above [9], [15], [16]. These data have confirmed that PrP^Sc^ typing in humans provides a surrogate molecular signature for certain prion strains within a particular host genotype, particularly when combined with neuropathological data.

Interestingly, PrP^Sc^ types 1 and 2 have also been found to co-exist in the same brain, often resulting in mixed clinical and neuropathological phenotypes. This phenomenon is referred to as “co-occurrence" and constitutes yet another variable in the factors that govern phenotypic variability. Although earlier studies have argued that co-occurrence of PrP^Sc^ types is mainly a function of the extent of brain sampling and the sensitivity with which a minority type can be detected in the presence of larger amounts of the other protein [17]–[22], two recent studies have placed firm restriction on the prevalence of cases with mixed PrP^Sc^ types and proposed that they constitute defined subtypes of sCJD with distinct clinico-pathological phenotypes [23], [24]. Nevertheless, debate on its extent and biological basis continues [25]–[27]. So far, information of the incidence of pure and mixed sCJD subtypes has not yet been provided comprehensively for single countries or areas.

National Surveillance Programs were initiated in many countries around the world to monitor the incidence of both existing and emerging forms of prion diseases, in particular new variant CJD (vCJD), which is causally related to bovine spongiform encephalopathy (BSE) in cattle [28]. In doing this, surveillance programs have in many ways served their purpose, as judged by the recent identification of variably protease sensitive prionopathy (VPSPr), a novel form of sporadic prion disease [29]. In the Netherlands, a National CJD Surveillance Program was established in 1997 to undertake prospective surveillance. In this report, we describe the epidemiological, clinical and neuropathological results of our comprehensive surveillance of prion diseases over a 12 year period, combined with genetic studies and prion protein characteristics.

## Methods

### Case selection and clinical evaluations

This study was embedded within collaborative studies monitoring the incidence of CJD in several European countries, using identical methodology. In the Netherlands, the National Prion Disease Registry, part of the Erasmus University Medical Centre in Rotterdam, aims to ascertain all patients with *definite* or *probable* CJD. Treating physicians, mainly neurologists, are required by law to notify all clinically suspected patients within two days to the reference centre. Whenever possible, all referred cases were classified before death by a member of the National Prion Disease Registry according to international criteria for *probable* or *possible* CJD or *other disease*, as described previously [2], [30]. For patients reported after death, classification was based on evaluation of symptoms and signs that could be retrieved from the hospital records.

Post mortem examinations, if permission was available, were carried out in the Dutch Surveillance Centre for Prion Diseases, part of the University Medical Centre in Utrecht. Local ethical committee approval was obtained for research on retained tissues after written informed consent given by the patients during life or their next of kin after death (Medical Ethics Committee of the University Medical Centre Utrecht 11-531/C). All information was analysed anonymously. The present study was restricted to patients that had been evaluated during the calendar years 1998–2009, as detailed clinical or neuropathological and biochemical data were not available for cases that had been referred before that time. When available, *PRNP* codon 129 genotypes from *probable* cases (i.e. the patients who had not given permission for autopsy) were analysed as well.

Case subjects underwent detailed evaluation using all available medical and risk-related information, obtained from both medical records, attending medical care providers and family interviews. The clinical features were reviewed prospectively with special attention given to initial symptoms, age at onset, disease duration and typical CJD symptoms (dementia, ataxia, pyramidal and extrapyramidal signs, myoclonus and akinetic mutism). Disease onset was calculated starting from the presentation of neurological signs or symptoms suggestive of organic involvement. Prodromal symptoms, such as tiredness, depression, sleep disturbance, abnormal appetite, weight loss and headache were not considered. Disease duration was counted as time from disease onset to death. A family history for neurodegenerative disease or a history of potential iatrogenic exposure (e.g. from dura mater implants, corneal grafts, or human cadaveric pituitary hormones) was assessed in all cases.

### Neuropathology

Brains were removed at autopsy and selected samples of tissue from the temporal cortex, occipital cortex and cerebellum were immediately frozen and stored at −80°C. The rest of the brain was fixed in formalin and used for histological and immunohistochemical purposes. Histopathological examination was performed on 5-µm-thick sections of formalin-fixed and paraffin-embedded brain tissue blocks, after decontamination for 1 hour in concentrated formic acid (98%). Sections were taken from the following areas: frontal (superior and middle frontal gyri), parietal (superior and middle parietal gyri), temporal (middle temporal gyrus) and occipital (calcarine cortex) cortices, hippocampus (Ammon's horn) with trans(entorhinal) cortex, striatum (caudate nucleus, putamen and globus pallidus), thalamus (anterior and pulvinar), brainstem (midbrain including periaqueductal gray, pons including locus coeruleus and medulla oblongata including inferior olive nucleus) and cerebellum (vermis and hemisphere). Haematoxylin-eosin and combined Luxol fast blue-periodic acid-Schiff (PAS) stains were performed according to standard procedures. The monoclonal antibody 3F4 (1∶400, overnight at 4°C, Signet Labs, MA, USA) was used for PrP immunohistochemistry. Pretreatment protocols for PrP staining involved antigen retrieval by autoclaving in citric acid buffer pH 6.0 at 121°C for 10 minutes followed by incubation with Proteinase K (10 µg/mL, for 5 minutes at room temperature). Evaluation of spongiform change and immunohistochemical PrP deposits was carried out in all cases of neuropathologically confirmed prion disease by comparing Luxol fast blue-PAS sections from 17 brain regions.

### Genetic analysis

Genomic DNA from 161 subjects with neuropathologically confirmed prion disease was extracted from blood or frozen brain tissues and used to amplify the coding region of *PRNP* in the polymerase chain reaction (PCR), as previously described [31]. The PCR products were visualized on 1% agarose gels to detect potential insertions or deletions. Potential point mutations were revealed by denaturing high pressure liquid chromatography (dHPLC) analyses. In 60 patients (37.2%), mutations were also ruled out by direct sequencing of the *PRNP* open reading frame (ORF). The codon 129 genotype was examined by digestion with the restriction endonuclease *Nsp* 1. All genetic analyses were performed at the Dipartimento di Scienze Neurologiche, Università di Bologna, Italy, except for six cases with a positive family history that had already been analysed by the Genome analysis department of the Academic Medical Centre in Amsterdam, the Netherlands. Information about the distribution of the codon 129 M/V polymorphism in the normal Dutch population was obtained from a previous study [32] and provided by the National Prion Disease Registry in Rotterdam, the Netherlands. The same institution also provided data on the codon 129 polymorphism in 32 *probable* CJD patients of whom blood was available for genetic analysis.

### Prion protein analysis

Preparation of samples, Western blotting and PrP^Sc^ typing were performed according to established methods [24], [26], [33], [34]. Two or three brain regions were examined, including the temporal cortex, occipital cortex and/or cerebellum. All samples were homogenized in lysis buffer plus [100 mmol/l NaCl, 10 mmol/l EDTA, 0.5% (v/v) Nonidet P40, 0.5% (w/v) sodium deoxycholate, 100 mmol/l Tris-HCL) at pH 6.9, digested with proteinase K (PK) (Roche Diagnostics, specific activity by certificate of analysis: 47.9 U/mg) at a final concentration of 10 U/ml and run in a 6.5 cm long separating gel. All immunoblots were probed with the monoclonal antibody 3F4 with epitope at PrP residues 108–111 at a 0.1 µg/ml concentration (Signet Labs, MA, USA). If immunohistochemistry had shown a mixed pattern of PrP^Sc^ deposition, whereas no evidence for or only scanty and focal type 2 accumulation had been found during immunoblotting, blots were also probed with the monoclonal antibody 1E4 with epitope at PrP residues 98–100 at 2 µg/ml concentration (Sanquin Reagents, Amsterdam, the Netherlands) as described [24]. All immunoblots were performed at the Dipartimento di Scienze Neurologiche, Università di Bologna, Italy.

### sCJD subtype classification

sCJD cases were classified according to Parchi *et al.* [24] based on the combined results of neuropathological examination, prion protein isotyping, and codon 129 genotyping. This updated classification system includes six “pure" subtypes of sCJD that are largely defined by the PrP^Sc^ type/codon 129 genotype combination and identified as (1) MM1/MV1; (ii) VV2; (iii) MV 2K; (iv) MM 2C; (v) MM 2T; and (vi) VV1 [14], and at least 4 additional “mixed" subtypes (MM/MV 1+2C, MM/MV 2C+1, VV 2+1, MM/MV 2 K+C) showing the co-occurrence of phenotypic features of two “pure" subtypes in the same brain. In the present study, the finding of mixed features at histopathological examination or the co-occurrence of PrP^Sc^ types 1 and 2 on western blotting was considered sufficient to attribute a case to a “mixed" subtype. In brief, the pathological phenotype of the MM/MV 1+2C largely overlaps with that of pure MM/MV 1 cases but also shows a) large vacuoles, often forming grape-like confluent foci of spongiform changes in addition to the classic microvacuolation of the MM1/MV1 subtype and b) a mixed synaptic/perivacuolar or coarse pattern of PrP staining. The pathological phenotype of MM/MV 2C+1 largely corresponds to that of the MM/MV 2C subtype, with the exception of a synaptic pattern of PrP staining in the cerebellum. The MV 2 K+C subtype is characterized by type “2C" pathology (as described above for MM/MV 1+2C) superimposed on the classic MV 2K lesion profile. And finally, the VV2+1 subtype can only be diagnosed by PrP^Sc^ typing, since the pathological phenotype in these subjects is very similar to that of VV2 cases.

### Statistical analysis

Means, tests of normality, χ^2^-tests and Kruskal-Wallis tests were performed using the Statistical Package for the Social Sciences package version 16.0 (SPSS Inc.). Statistical significance was defined as *P*<0.05. Incidence was calculated as the number of newly detected *definite* (e.g. pathologically confirmed) and *probable* TSE cases per year per million inhabitants. Since prion diseases are invariably fatal and illness duration is usually less than one year, the incidence and mortality were considered equal. Denominator population data for the years 1998–2009 were provided by the Department of Epidemiology of the Erasmus University in Rotterdam, The Netherlands.

## Results

### All patients with clinical suspicion of prion disease

Between January 1998 and December 2009, 451 patients with a clinical suspicion of prion disease were registered by the Dutch National Prion Disease Registry in Rotterdam. Of these, 295 patients were notified prospectively and 156 patients were reported after death. Most of the latter were referred directly for post mortem examination to the Dutch Surveillance Centre for Prion Diseases in Utrecht without central registration in Rotterdam. Overall, 318 of the 451 registered patients with suspected prion disease underwent post-mortem examination, equalling an autopsy rate of 70.5%. The relationship between clinical classification and post-mortem results in this group is provided in **Table 1**. Total autopsies averaged 27 per year (**Table 2**). A *definite* diagnosis of prion disease was confirmed in 162 patients (50.9%) and in 156 patients (49.1%) neuropathology failed to detect prion disease. Overall, of all patients with neuropathologically confirmed prion disease (*n* = 162), 134 patients (82,7%) were notified prospectively. The remaining 28 patients (17,3%) were reported after death. There were 52 patients classified as *probable* CJD among the 133 referred cases in which no permission for autopsy was obtained. Blood and/or frozen tissues were available for further biochemical and genetic studies in 161 patients with *definite* prion disease. There were 9 patients (5.6%) in whom *PRNP* genotyping confirmed a genetic form of TSE. The total annual mortality of TSE in the Netherlands has remained more or less stable (*P* = 0.06, χ^2^-test) over the observation period, ranging from 0.63 to 1.53 per million inhabitants per annum (**Table 2**).

**Table 1:** Patients sorted by clinical classification with autopsy results.

		Autopsy results	
Clinical classification	Total (n)	Definite CJD (n)	Non-CJD (n)
Clinical classification as	161 (50.6%)	127 (78.4%)	34 (21.8%)
“probable CJD"			
		sCJD (n = 119)	Alzheimer/CAA (n = 6)
		gCJD (n = 2)	Multi-infarction (n = 5)
		iCJD (n = 4)	Alzheimer/Alzheimer changes (n = 4)
		vCJD (n = 2)	Auto-immune encephalopathy (n = 6)
			Lewy Body Dementia (n = 3)
			Non-Hodgkin Lymphoma (n = 2)
			Alzheimer/infarction (n = 2)
			Frontotemporal Dementia (n = 1)
			Metastasis (n = 1)
			Pinealoblastoma (n = 1)
			Unknown aetiology/other (n = 3)
Clinical classification as	49 (15.4%)	19 (11.7%)	30 (19.2%)
“possible CJD"			
		sCJD (n = 16)	Alzheimer/Alzheimer changes (n = 7)
		gCJD (n = 1)	Lewy Body Dementia (n = 6)
		VPSPr (n = 1)	Auto-immune encephalopathy (n = 4)
		iCJD (n = 0)	Frontotemporal Dementia (n = 2)
		vCJD (n = 1)	Progressive Multifocal Leuko-encephalopathy (n = 2)
			Alzheimer/CAA (n = 2)
			Non-Hodgkin Lymphoma (n = 1)
			Metastasis (n = 1)
			Alzheimer/infarction (n = 1)
			Multi-infarction (n = 1)
			Viral encephalitis (n = 1)
			Unknown aetiology/other (n = 2)
Clinical classification as	108 (33.9%)	16 (9.9%)	92 (59.0%)
“other disease"			
		sCJD (n = 9)	Alzheimer/Alzheimer changes (n = 21)
		gCJD (n = 6)	Multi-infarction (n = 13)
		iCJD (n = 1)	Alzheimer/infarction (n = 9)
		vCJD (n = 0)	Lewy Body Dementia (n = 7)
			Auto-immune encephalopathy (n = 6)
			Infectious (n = 5)
			Alzheimer/CAA (n = 4)
			Frontotemporal Dementia (n = 3)
			Non-Hodgkin Lymphoma (n = 3)
			Metastasis (n = 2)
			Multiple sclerosis (n = 2)
			Spinocerebellar ataxia (n = 1)
			Amyotrophic lateral sclerosis (n = 1)
			Toxic metabolic (n = 1)
			Unknown aetiology/other (n = 14)
	318 (100%)	162 (100%)	156 (100%)

CJD = Creutzfeldt-Jakob disease; sCJD = sporadic Creutzfeldt-Jakob disease; gCJD = genetic Creutzfeldt-Jakob disease; iCJD = iatrogenic Creutzfeldt-Jakob disease; vCJD = variant Creutzfeldt-Jakob disease; VPSPr = variably protease-sensitive prionopathy; CAA = cerebral amyloid angiopathy.

**Table 2:** Mortality and classification of all *definite* and *probable* TSE patients.

Year of surveillance		1998	1999	2000	2001	2002	2003	2004	2005	2006	2007	2008	2009	total
Notified prospectively		40	46	41	32	36	32	37	37	46	37	38	29	451
Referred for post-mortem examination		25	28	20	18	26	19	26	32	38	30	33	23	318
Total number of patients with TSE*		18	20	10	17	18	13	22	23	25	17	18	13	214
Aetiology of **definite** TSE														
	Sporadic CJD	12	16	6	8	11	7	13	17	19	10	15	10	144
	Iatrogenic CJD	1	1	0	1	0	1	1	0	0	0	0	0	5
	Inherited CJD	0	0	0	0	0	0	0	0	1	2	0	0	3
	GSS	0	0	0	1	0	0	1	1	1	0	0	0	4
	PrP-CAA	0	0	0	0	0	0	0	0	0	0	1	0	1
	FFI	0	0	0	0	0	0	0	0	0	0	1	0	1
	Variant CJD	0	0	0	0	0	0	0	1	1	0	0	1	3
	VPSPr	0	0	0	0	0	0	0	1	0	0	0	0	1
Subtotal		13	17	6	10	11	8	15	20	22	12	17	11	162
Mortality TSE**		1.20	1.30	0.63	1.07	1.13	0.81	1.34	1.41	1.53	1.04	1.10	0.78	
Mortality sCJD**		1.13	1.24	0.63	0.88	1.13	0.75	1.22	1.22	1.34	0.92	0.98	0.66	

TSE = Transmissible Spongiform Encephalopathy; CJD = Creutzfeldt-Jakob disease; sCJD = sporadic Creutzfeldt-Jakob disease; GSS = Gerstmann-Sträussler-Scheinker disease; PrP-CAA = prion protein cerebral amyloid angiopathy; FFI = Fatal Familial Insomnia; VPSPr = variably protease-sensitive prionopathy.

* Definite and probable cases.

** Per million inhabitants per annum.

### Patients with sCJD

A *definite* diagnosis of sCJD was made in 144 patients (88.9% of all patients with *definite* prion disease). The mean age at onset was 67 years (range 35 to 85 years) and the mean disease duration 6.5 months (range 1 to 36 months). In this group, 62 patients (43.0%) were males and 82 patients (57.0%) females. Genetic analysis for the codon 129 MV polymorphism in all *definite* sCJD cases (available in *n* = 143) revealed 101 (70.6%) MM cases, 32 (22.4%) MV cases and 10 (7.0%) VV cases (**Table 3**). The sCJD codon 129 distribution in The Netherlands is comparable to the sCJD distribution in the surrounding Western European countries [14], [35] (*P* = 0.05, χ^2^-test), although there was a trend for under-representation of VV cases (*P* = 0.06).

**Table 3:** Codon 129 distribution in the normal Caucasian and Dutch population and in sCJD patients [32], [35], [46].

Codon 129	Normal Western European population (%)	Normal Dutch population (%)	Sporadic CJD in Western Europe (%)	Sporadic CJD in The Netherlands (%)
MM	44.1	45.1	71.0	70.6
MV	44.5	45.6	13.0	22.4
VV	11.4	9.3	16.0	7.0

M = methionine; V = valine.

Frozen tissue was available for immunoblotting in 140 of 144 patients with neuropathologically confirmed sCJD. Molecular typing by western blotting showed that 71 cases had only PrP^Sc^ type 1, 33 only PrP^Sc^ type 2 and 36 cases the presence of both PrP^Sc^ types 1 and 2 (**Table 4**). Similar to previous studies [24], the PrP^Sc^ type 1 and 2 protein mixture was detected in each subtype, but more frequently in MM than in MV or VV cases. Finally, 3 cases showed a distinctive atypical profile characterized by truncated protein fragments of relatively low molecular weight on immunoblotting or histopathological features that were not consistent with any of the specified molecular subgroups.

**Table 4:** *PRNP* genotype and PrP type in 140 sCJD patients.

Type	MM	MV	VV	Total
Type 1	63* (64.3)	8 (25.0)	0 (0)	71 (50.7)
Type 1+2 concurrence	32 (32.6)	2 (6.3)	2 (20.0)	36 (25.7)
Type 2	3 (3.1)	22 (68.7)	8 (80.0)	33 (23.6)
Total	98	32	10	140

MV 1+2 are 2 cases without Kuru plaques, MM2 includes 1 MM 2C and 2 MM 2T cases, whereas MV2 includes 14 MV 2K cases and 8 MV 2 K+C cases.

% are expressed in brackets and refer to each single column.

• in 13 cases the histopathological findings suggested the additional presence of type 2.

Cases were classified according to Parchi *et al.* [24] based on molecular features and neuropathological lesion profiles, and their subgroup allocation is shown in **Table 5**. A pure phenotypic subtype was found in 60.1% of the sCJD patients, whereas mixed phenotypes (determined by either PrP^Sc^ or histopathological typing or both) were observed in 39.9% of patients. The subtype allocation was based on results of both neuropathological examination and prion protein isotyping in 127 patients (i.e. the results of both examinations were compatible). In 13 patients, neuropathological examination identified the distinctive features of the MM/MV 2C subtype despite the absence of a type 2 band on immunoblotting, while the two VV 2+1 cases were recognized by PrP^Sc^ immunoblotting. Similar to earlier results, the various subgroups showed differences in disease duration (*P*<0.01, Kruskal-Wallis test). Mean disease duration stratified by molecular subgroup was the shortest for MM/MV 1 with 3.0 months (range 1–8) and longest for the MV 2 K+C and MM/MV 2C+1 subgroups with 15.6 months (range 4–36 months, excluding single cases in the MM 2C and MM 2T subgroups) (**Table 5**).

**Table 5:** Classification of sporadic CJD subtypes in the Dutch population based on combined molecular and histopathological assessment.

	No. cases	%	Mean age at onset (years)	Mean duration (months)
**Pure subtypes**	**83**	**58**		
MM1/MV1	58	40.6	67 (42–85)	3 (1–8)
VV2	8	5.6	72 (61–80)	7.9 (3–24)
MV 2K	14	9.1	63 (53–74)	13.5 (3–36)
MM/MV 2C	1	<1	69	16
MM 2T	2	1.4	47 (35–58)	26 (18–34)
**Mixed subtypes**	**57**	**39.9**		
MM/MV 1+“2C"	42*	29.4	68 (50–82)	4.0 (1–15)
MM/MV 2C+1	5	3.5	66 (54–73)	15.6 (12–21)
VV 2+1	2	1.4	59	6
MV 2 K+C	8	5.6	68 (57–75)	15.6 (4–36)
**Atypical**	**3**	**2.1**	67 (63–74)	12.7 (10–16)

Nomenclature is largely based on the codon 129 genotype, which can be either methionine (M) or valine (V) and the PrP^Sc^ type (type 1 or 2, according to Parchi *et al.*) Since both MM2 and MV2 groups are associated to 2 distinct phenotypes, these are further defined with a third parameter (*capital letter*) referring to distinctive histopathological features: *K* kuru type amyloid plaques, *C* predominant cortical pathology with confluent vacuoles and perivacuolar PrP staining, *T* prominent thalamic pathology with atrophy.

* In 13 patients, neuropathological examination identified the distinctive feature of the MM/MV 2C subtype (i.e. grape-like clusters of spongiform change with a coarse PrP immunohistochemical staining pattern), but the type 2 band on immunoblotting was not detected.

In total, 70.6% of patients with sCJD died within 6 months of illness onset, and 53.8% died within 3 months. A disease duration of more than 24 months was found in 5 sCJD patients (3,5%). Interestingly, four of them belonged to the MV 2K or MV 2 K+C subgroups. Although previous reports have suggested that long disease duration, especially in Japanese sCJD patients, may reflect differences in the clinical management, this is likely not the case in our cohort of patients, as ventilation and life-sustaining treatment are only seldom used in patients with CJD in Europe. Altogether, four sCJD patients (two MM1, one MV1 and one MM 2T) with age at onset below 50 years were examined, the youngest patient at the age of 35 years. The age group over 80 years at onset consisted of 8 patients (5.6%), all within the MM1 and MM 1+2C subgroups.

### Patients with genetic prion disease

Within the group of patients with *definite* prion disease available for mutation analysis, 9 patients (5.6%) were positive for a *PRNP* mutation. All patients with the same mutation belonged to the same family. These patients had been analysed on the basis of positive family history or unusual clinical and neuropathological features. Genetic analysis for *PRNP* revealed 4 patients with GSS, 3 patients with gCJD, 1 patient with PrP cerebral amyloid angiopathy and 1 patient with FFI (**Table 6**). Several patients were already published as case reports [36]–[40]. Genetic analysis of the remaining 152 patients with *definite* prion disease revealed eight A117A, 129V polymorphisms (4.9%), two DelR3/R4, 129M polymorphisms (1.2%) and one G124G, 129M polymorphism (0.6%), but no pathogenic mutations. This was confirmed by direct sequencing of the *PRNP* ORF in 51 (33.5%) cases.

**Table 6:** Demographic characteristics of patients with genetic CJD (1998–2009), caused by various mutations in *PRNP*.

Mutation	age at onset (years)	Duration (months)	Codon 129 (Met/Val)	PrP^Sc^ type	Family history	Clinical features	Pathological features
ins 168 bp, 129V	51–59	7–65	VV and MV	1+8 kDa	positive	Dementia, extrapyramidal, cerebellar signs	GSS
G131V, 129M	36	188	MV	8 kDa	positive	Cognitive decline, ataxia, parkinsonism	GSS with neurofibrillary tangles
ins 120 bp, 129M	35	92	MM	1+2	positive	Dementia, extrapyramidal signs, personality change	CJD with abnormal Purkinje cell migration
D178N, 129V	49–50	24–42	MV	1	positive	Dementia, ataxia, myoclonus, (extra)pyramidal signs	CJD
Q227X, 129V	39	72	MV	7 kDa	positive	Dementia, extrapyramidal signs	GSS with neurofibrillary tangles
Y226X, 129V	55	27	MV	n.a.	positive	Dementia, visual and acoustic hallucinations	PrP-CAA with tau deposits
D178N, 129M	58	7	MM	2b	positive	Insomnia	FFI with tau deposits

All Ins = insertion; bp = base pairs; M = methionine; V = valine; n.d. = not determined;

CJD patients with the same mutation belong to the same family.

= Creutzfeldt-Jakob disease; GSS = Gerstmann-Sträussler-Scheinker disease; FFI = Fatal Familial Insomnia; PrP-CAA = prion protein cerebral amyloid angiopathy.

### Patients with iCJD

During the 11 years of surveillance, we identified 5 patients with iatrogenic CJD (4 patients due to dura mater grafts and 1 patient after hGH injections) (**Table 7**). The disease duration ranged from 2 to 9 months. The dura mater patches were performed between 1982 and 1988. In all patients Lyodura® was used, produced by Melsungen AG in Germany. Incubation time varied from 10 to 21 years (mean 16.0 years). Two of these patients showed a VV1 molecular subtype, whereas the other two patients showed a MM1 and MV1 subtype, respectively. The hGH injections were given in 1963 as part of a diagnostic process. The original supplier could not be retrieved anymore from the hospital records. This patient developed CJD at the age of 47 years, after an incubation period of 38 years, the longest ever reported [41]. The disease duration was 6 months and on immunoblotting he showed a MM1 subtype.

**Table 7:** Demographic characteristics of all patients with iatrogenic CJD (1998–2009).

Year of death	Age at onset (years)	Disease duration (months)	Incubation period (years)	PrP^Sc type^	Codon 129 (M/V)	Mode
1998	51	6	14	1	VV	Dura mater graft
1999	54	4	10	1	MM	Dura mater graft
2001	47	6	38	1	MM	HGH injections
2003	66	9	21	1	MV	Dura mater graft
2004	65	2	19	1	VV	Dura mater graft

hGH = human Growth hormone; M = methionine; V = valine.

### Patients with vCJD

Between 1998 and 2009, three patients with vCJD were diagnosed. The first patient was identified in 2005 [42], the other two were reported in 2006 and 2008. Patient characteristics are provided in **Table 8**.

**Table 8:** Demographic characteristics of all patients with new variant CJD.

Year of diagnosis	Age at onset (years)	Disease duration (months)	Codon 129 (M/V)	Symptoms at disease onset
2005	24	20	MM	anxiety, forgetfulness
2006	14	15	MM	concentration disturbance
2009	48	14	MM	depression, anxiety

M = methionine; V = valine.

### Patient with VPSPr

Recently, Gambetti *et al.* identified a novel form of variably protease-sensitive prionopathy (VPSPr), characterized by the presence of an abnormal PrP species that was largely sensitive to PK digestion [29], [43]. After re-evaluating cases with atypical dementias in the Dutch surveillance program, we were able to identify one case of VPSPr in a patient was homozygous for valine at codon 129 of *PRNP*. This patient was reported as a case study [44].

### Patients with other disease

In 156 patients, neuropathology failed to detect any evidence of prion disease. In 36.0% of these cases, another neurodegenerative disease was neuropathologically confirmed, such as Alzheimer's disease (21.2%), Lewy body dementia (9.6%) and frontotemporal lobar degeneration (3.8%) (**Table 9**). A substantial group (∼14.0%) suffered from a potentially treatable disorder such as infectious disease, tumour-associated disease or toxic/metabolic disorders. Vascular disease (∼20%) and autoimmune encephalopathy (10,3%) were responsible for most remaining cases. Thirteen patients (8.3%) had co-existent pathology in the form of a vascular event superimposed on Alzheimer's disease, explaining the rapid deterioration in the later stages of the disease course.

**Table 9:** Autopsy results in all patients with alternative diagnoses.

Condition	Cases (n)	Percentage of non prion disorders
**Neurodegenerative disease**	56	35.9
Alzheimer/Alzheimer changes	33	
Lewy body dementia	15	
Frontotemporal lobar degeneration	6	
Spinocerebellar ataxia	1	
Amyotrophic lateral sclerosis	1	
**Immunologically mediated**	18	11.5
Auto-immune encephalopathy	16	
Multiple sclerosis	2	
**Infectious**	8	5.1
Progressive multifocal leukoencephalopathy	2	
Herpes simplex virus	1	
atypical mycobacteria	1	
Human immunodeficiency virus	1	
Lues	1	
Other	2	
**Malignancy**	12	7.7
Non-Hodgkin Lymphoma	6	
Metastasis	4	
Primary	2	
**Toxic/metabolic**	1	0.7
Acute Intermittent Porphyria	1	
**Vascular**	31	19.9
Multi-infarct dementia	19	
Cerebral amyloid angiopathy	12	
**Mixed Alzheimer disease/vascular**	13	8.3
**Unknown aetiology/other**	17	10.9
**Total**	156	100

## Discussion

This study analyses the results of National Surveillance for TSEs over a 12 year period within the population of 16 million inhabitants of the Netherlands since 1998. To our knowledge, this is the first time that epidemiological, genetic and molecular data, including information about the incidence of pure and mixed sCJD subtypes have been provided comprehensively for a single country. Most countries that have undertaken surveillance over an extended period of time have reported a steadily progressive increase in the recorded incidence of sCJD [3], [45]. This has been attributed to enhanced case detection and reporting. In this study, however, we found a more or less stable incidence and mortality from TSE and sCJD during the years 1998–2009, ranging from 0.63 to 1.53 per million inhabitants per annum.

The clinical and pathological phenotypes of our sCJD patients were fully compatible with the updated classification scheme proposed by Parchi *et al.* [14], [24], except for 3 atypical cases which defied classification. Codon 129 polymorphism analyses revealed a more or less similar genotype distribution compared to previously reported distribution in other cohorts [14], [35], with an increase of MM homozygotes (70.6%) compared to the normal population. Interestingly, although not statistically significant, the proportion of MV heterozygous sCJD patients tended to be higher than that reported in other studies [14], [35], [45], [46], whereas the VV homozygous patients were relatively underrepresented. Although the relatively high proportion of 129VV patients in our *probable* CJD group (i.e. 25% of a group of 32 tested cases), may, at least in part, compensate for the observed abnormal distribution in the *definite* CJD group, the finding is overall intriguing and deserves consideration and future follow-up. The codon 129 distribution in the normal Dutch population is comparable to other Western European countries [46]–[48].

An important aspect of sCJD phenotypic variability concerns the existence of cases presenting more than one PrP^Sc^ type and/or mixed histopathological features in the same brain. In previous studies, the proportion of cases with concurrent PrP^Sc^ types varied between 12 and 44% and was believed to be a function of the sampling protocol that was employed [14], [17]–[22]. Recently, however, Parchi *et al.* carried out a systematic study in a large series of Italian, German and North-American sCJD patients and found the co-occurrence of characteristic features (either molecular or histopathological or both) of two distinct sCJD subtypes in about 40% of cases [24]. For a reliable sCJD group classification, the authors recommended a protocol including the neuropathological assessment of at least eight brain regions and PrP^Sc^ typing in four critical regions, such as the temporal, parietal and occipital neocortices, and medial thalamus. By applying this protocol with some modifications (e.g. only two of the four recommended brain regions were available for typing), we performed group classification for the Dutch sCJD patients. The results were largely compatible with the updated classification by Parchi *et al.* [24]. A mixed sCJD subtype, inferred from either neuropathological examination, the results of immunoblotting or both analyses, was again found in about 40% of patients and was higher in MM than in MV or VV subjects. In the present study, neuropathological examination was more sensitive than PrP^Sc^ typing in identifying the distinctive features of the MM/MV 2C subtype when associated focally to the MM/MV1 subtype, the result being at least partially explained by the relative low number of brain areas available for PrP^Sc^ typing. Our observations reinforce the usefulness of the proposed protocol for typing of sporadic CJD cohorts and underline the necessity of careful histopathological evaluation, particularly when the availability of frozen tissue for PrP^Sc^ typing is limited.

In contrast with this and other studies [23], [24], [26], [49], others [25], [50] have suggested that it may not be possible to place firm restrictions on the prevalence of cases with mixed PrP^Sc^ types. By using type-1 [25] and type-2 [50] selective antibodies, these studies detected, in little amounts, type-1 PrP^Sc^ in all patients with sCJD type 2 and type-2 PrP^Sc^ in all patients with sCJD MM1. Although the interpretation and relevance of these data for CJD classification and strain-typing was contested [26], [49], by showing that such PrP^Sc^-selective antibodies recognize PrP fragments that do not match the physicochemical properties of those detected with standard PrP^Sc^ typing, an issue that was also discussed in the latter of these studies [50], these results are of interest and further investigations are required to unravel the biological basis of co-occurrence of PrP types.

In the Netherlands, only 5 iatrogenic CJD cases were identified over a 12 year period, corresponding to a lower incidence (3,1%) than for other countries with CJD surveillance [51]. Similarly, genetic analysis of *PRNP* in 162 patients with neuropathologically confirmed prion disease revealed a mutation in only nine of them, corresponding to an incidence of 5,6%. This figure is slightly lower then the average rate of inherited prion diseases in Europe (9,5%) [45], but higher than the previously reported 2% for the Netherlands [30] and comparable to the figures from neighbouring Germany [45]. The slightly lower rate of inherited prion disease in the Dutch population cannot be a result of underdetection of *PRNP* mutations, since all cases with *definite* prion disease were genotyped in this study. Interestingly, some of the most frequent mutations (e.g. P102L, E200K and V210I) in Caucasian populations, including those from the surrounding Western European countries [30], [45], were not identified in the Netherlands, possibly related to the relatively small study group. Other than the codon 129 MV polymorphism, the two most common non-pathogenic *PRNP* variants in the Dutch population were A117A (4.9%) and a 24 bp deletion in the octapeptide repeat region (1.2%). These data are comparable to other studies [46].

Many reports on patients with clinical syndromes mimicking CJD are found in the literature with a broad range of diagnoses. Analysis of differential diagnosis revealed Alzheimer's disease as the most frequent other diagnosis among our patients (21.2%). This is not surprising, as it represents the most frequent cause of dementia in the elderly. Other diagnoses such as immunologically mediated dementia, multi-infarction dementia and neoplastic disease also presented with a clinical syndrome similar to that of prion disease. Many of these patients were referred to the National Prion Disease Registry on the basis of a false-positive 14-3-3 test in cerebrospinal fluid, myoclonus, akinetic mutism or an accelerated decline in the later stages of disease, most often caused by superimposed vascular disease (e.g. infarction). The high number of non-TSE cases in our study (∼50%) at least seems to indicate that the surveillance system in the Netherlands is very accessible to all treating physicians.

In conclusion, the incidence and mortality rates of sCJD in the Netherlands are similar to those in other European countries, whereas iatrogenic and genetic cases are relatively rare. The unusual incidence of the VV2 sCJD subtypes compared to that reported in other Western countries deserves further investigation.
